# Abundance–Occupancy Relationships are Informed by Species Temporal Occupancy

**DOI:** 10.1002/ece3.73214

**Published:** 2026-04-06

**Authors:** Lauren A. Holian, Cleber Ten Caten, Tad A. Dallas

**Affiliations:** ^1^ Department of Biological Sciences University of South Carolina Columbia South Carolina USA; ^2^ Department of Botany University of Wisconsin Madison Wisconsin USA

**Keywords:** abundance–occupancy, macroecology, mammals, spatiotemporal relationships

## Abstract

Understanding the relationship between species occupancy and abundance is a central goal in macroecology. The positive link between species spatial occupancy—the fraction of sites in which a species is present—and mean local abundance has been dubbed a ‘macroecological law’. However, this relationship fails to capture changes in occupancy over time. Temporal occupancy—estimated as the fraction of times a species is recorded in each site—has been used to explore species persistence in local communities but has no clear relationship with mean local abundance. That is, do more persistent species tend to occur at higher abundance? Further, how might species temporal occupancy relate to their spatial occupancy? We explored the relationship between spatial and temporal occupancy, and their respective relationships with mean local abundance using standardized sampling data of small mammal communities from the National Ecological Observatory Network (NEON). These sampling data spanned across the United States from 2014 to 2019. Except for several range‐restricted species, species that occupied a greater fraction of sites also tended to occupy individual sites more frequently. Additionally, species that were more widespread (high spatial occupancy) and/or more persistent (high temporal occupancy) tended to occur with greater local abundance. However, the highest local abundance was observed for geographically restricted but temporally persistent species. This indicates that while there is support for the positive abundance‐spatial occupancy relationship, spatial occupancy alone may underestimate the abundance of range restricted species. In such cases, high local abundance may be better explained by the temporal frequency in which species occupy sites. Taken together, these results demonstrate the positive relationship between spatial and temporal occupancy and highlight the utility of considering temporal occupancy alongside spatial occupancy for understanding abundance patterns.

## Graphical abstract

Understanding how species spatial and temporal distributions are related to abundance is fundamental to ecology. Indeed, the relationship between species spatial occupancy and abundance has been investigated across taxa and environments. Less studied, is how species temporal occupancy may inform the spatial abundance–occupancy relationship. Here, we explore the relationship between species spatial occupancy, temporal occupancy, and mean local abundance, using small mammal communities sampled over time as part of the National Ecological Observatory Network.

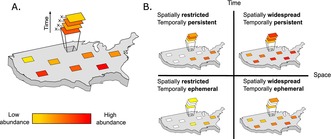

## 1 Introduction

Understanding how species spatial and temporal distributions are related to abundance is fundamental to ecology. Indeed, the positive relationship between species spatial occupancy and local abundance has been called a macroecological law (i.e., “abundance–occupancy” relationships) (Lawton 1999). However, while positive abundance–occupancy relationships are frequently observed across taxa and environments (Gaston et al. 2000; Ten Caten et al. 2022a; Verberk et al. 2010; Miranda and Killgore 2019, but see [Ferenc et al. 2016; Komonen et al. 2009]), they tend to be weak (Ten Caten et al. 2022a), and have a demonstrated sensitivity to spatial and temporal scales (Steenweg et al. 2018; Dallas et al. 2019). Despite this, abundance–occupancy relationships have largely ignored differences in species temporal occupancy, or the fraction of time in which species are present across sites (but see [Magurran and Henderson 2003; Soininen and Heino 2005; Magurran 2007]). Incorporating species temporal occupancy could be particularly pertinent for species whose high local abundance is not well explained by being spatially widespread, such as some habitat specialists (Verberk et al. 2010).

Previously, spatial and temporal occupancy have been used to characterize species as either spatially “core”/“satellite” (Hanski 1982) and as temporally “core”/“transient” (Magurran and Henderson 2003; Coyle et al. 2013; Snell Taylor et al. 2020). These frameworks are useful for conceptualizing how species tend to spatially and temporally occupy sites, but are not directly comparable. The temporal core/transient framework assesses occupancy at a single site (either from the fraction of years (Coyle et al. 2013; Snell Taylor et al. 2020; Hansen and Carey 2015; Wilfahrt et al. 2021) or months [Soininen and Heino 2005; Boss and Da Silva 2014]) whereas the spatial core/transient framework evaluates occupancy across sites (Coyle et al. 2013; Supp et al. 2015). Therefore, the core/transient framework allows a single species to be variably designated as core or transient depending on the site and assemblage. It does not, however, consider how temporal occupancy varies between species, how temporal occupancy relates to spatial occupancy, or how it relates to local abundance.

**FIGURE 1:**
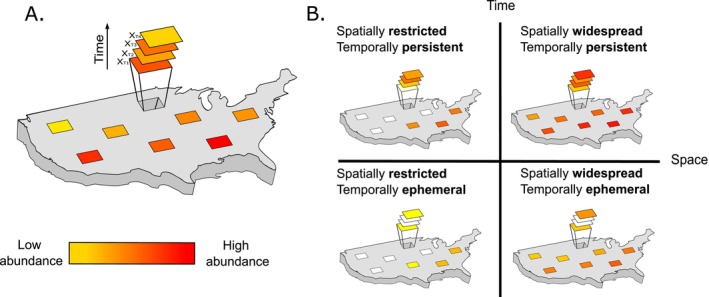
Abundance–occupancy relationships often focus on spatial occupancy, but may be better informed by both species spatial and temporal occupancy. (A) Across a region, spatial occupancy is quantified as the fraction of sites (represented by rectangles) in which a species is detected and its temporal occupancy is measured as the fraction of months it was detected across the sites in which it occurs (T1–T4). (B) Taken together, species can be considered in a spatiotemporal framework where species range from spatially restricted to widespread, and from temporally ephemeral to persistent. Widespread and persistent species may be less susceptible to environmental stochasticity or changes in land use, especially if these properties positively relate to local abundance (indicated by color). By contrast, a restricted and ephemeral species may be at a demographic disadvantage and less able to track environmental changes, making it more susceptible to environmental stochasticity, land use change, or other perturbations.

To estimate spatial occupancy, temporal occupancy, and abundance for the same taxa requires data from standardized sampling efforts that are capable of sampling multiple sites at the same time. This has been a fundamental limitation to addressing both spatial and temporal occupancy simultaneously and in the general evaluation of macroecological relationships (but see (MacKenzie et al. 2003; Briscoe et al. 2021) for the incorporation of temporal dynamics into occupancy estimations), but efforts such as the National Ecological Observatory Network (NEON) have allowed large‐scale spatiotemporal questions to be explored. Using the NEON small mammal sampling data, we investigate the relationship between spatial occupancy, temporal occupancy, and abundance across an environmentally heterogeneous region (United States), spanning 20 ecoclimate domains. Temporal occupancy estimated across sites (hereafter “regional” temporal occupancy) builds on the site‐level core and transient framework and facilitates direct comparison to the spatial core/satellite framework. Further, regional temporal occupancy represents how species tend to persist across sites, rather than their temporal dynamics at a given site. Additionally, we consider temporal occupancy intra‐annually which departs from previous work aimed at estimating population persistence across years (Magurran and Henderson 2003; Coyle et al. 2013; Hansen and Carey 2015; Snell Taylor et al. 2020; Wilfahrt et al. 2021), but see (Guo et al. 2000; Soininen and Heino 2005; Boss and Da Silva 2014). Instead, intra‐annual temporal occupancy focuses on how species use sites throughout the year, that is, are species persistently or ephemerally occupying sites? In this framework, species can be characterized from spatially restricted to widespread according to spatial occupancy and from ephemeral to persistent (not formal population persistence where growth rate r>=0; [Case 1999]) according to temporal occupancy (Figure 1).

Extending abundance–occupancy relationships by considering the role of temporal occupancy, we explore the consistency of spatial and temporal abundance–occupancy relationships for small mammal communities sampled across the United States. This was done across species (interspecific abundance–occupancy relationships), as well as within each small mammal species (intraspecifically). This addresses two fundamentally different questions: (1) Do more widespread or more temporally persistent small mammals have higher abundance (interspecific)? and (2) does interannual variation fundamentally couple species spatial or temporal occupancy with species abundance (intraspecific)? Owing to the strength of standardized sampling programs such as NEON, we are able to directly link spatial and temporal occupancy to address if widespread species also tend to be more temporally persistent in communities. We found that spatial and temporal occupancy were positively related, indicating that broadly distributed species tend to occupy sites for greater portions of the year, with several notable exceptions. Furthermore, though the intraspecific relationships were variable, both spatial and temporal occupancy were positively correlated to local abundance across species. This means that species with broader distributions or greater temporal frequencies tended to also have greater local abundance. However, the largest abundances were associated with species that had high temporal occupancy and low spatial occupancy, suggesting that species which are locally adapted can also attain high abundance.

## 2 Methods

### 2.1 NEON Data Collection

In order to understand the relationships between spatial and temporal occupancy and abundance, we used the National Ecological Observatory Network (NEON) small mammal data (National Ecological Observatory Network (NEON) 2022), accessed through the neonUtilities *R* package. The data consisted of 127 species spanning 40 genera sampled between 2014 and 2019. The 46 sites are distributed across the continental United States and represent 20 ecoclimate domains (see [Meier et al. 2023] for more information on the rationale and the Supplement for a site map). Within each site, small mammals were sampled based on established NEON protocols (Kao et al. 2012; Thorpe et al. 2016; Meier et al. 2023), where 100 Sherman live traps were arranged in three to eight 90 × 90 m grids, depending on available area. Throughout the year, sampling was conducted 4–6 times per site. Each sampling session occurred within 10 days of the new moon and lasted for one or three nights. The repeated standardized procedure and relatively large distance between sites make these data ideal for an investigation of spatial and temporal processes.

### 2.2 Quantifying Abundance

Mean local abundance of each species was calculated per year as the average monthly count of a species across the sites in which they occurred. Monthly count was calculated as the summed unique captures standardized by trapping effort, or the total number of operational traps set within that month (traps that were disturbed, not set, or noted for unusual circumstances were not considered). Only individuals identified to the species/subspecies level were included. Individuals recaptured within a single month and those recaptured without a recorded identification number (tagID in the NEON data) were excluded. The mean monthly abundance for each site was calculated for a given year, and then the mean across sites was taken for each species/subspecies and log transformed for analyses.

### 2.3 Quantifying Species Occupancy

Spatial occupancy was quantified as the fraction of sites in which each species was detected within a year. Though the number of sites sampled was relatively constant, the intensity at which each site was sampled was more variable. A low threshold for site occupation was therefore set as at least one record of a species at a site in a given year. We acknowledge that calculating spatial occupancy in this manner assumes relatively even detection probability, both across sites and species. While advances have been made in occupancy modeling (see Supplement for spatial occupancy model fit/performance)—specifically around the direct quantification of detection probabilities (MacKenzie et al. 2017)—the uncertainty in abundance estimates of the NEON small mammals (e.g., [Parsons et al. 2023]) makes the incorporation of detection probability into estimates of population abundance infeasible (see Supplement for comparisons of abundance estimates). We attempt to explore this in the Supporting Information, in which we consider sampling efforts in between known detections of at least one individual to simply be ‘missed detections’ and consider them present when quantifying occupancy. This does not change our findings (see Supporting Information).

Temporal occupancy was quantified as the mean fraction of months a species was detected across sites for a given year weighted by the number of months each site was sampled. This meant that for a species‐year combination each site where it was detected was assigned a value of site‐level temporal occupancy (months present/months sampled) and then the weighted mean was taken across sites, accounting for differences in the amount of months each site was sampled (1–8 months of sampling at a site). This assumes that lack of detection represents species absence from a site, even if they were present in the preceding and/or following months. To ensure our results were robust to this assumption, we also ran the analyses assuming that species occupy sites for all sampled months between their first and last detection within a year and found that this did not substantially alter the relationships (see Supporting Information for further details).

Both spatial and temporal occupancy were calculated as a single landscape measure for a given species in a year in order to facilitate direct comparison between spatial and temporal occupancy. Additionally, considering abundance and occupancy at the annual scale focuses our study on more general demographic patterns than those that might be observed between seasons. However, the annual scale may not be appropriate for all taxonomic assemblages and could be sensitive to differences in life history (e.g., generation time) or phenology. To this end, we recalculated temporal occupancy as the fraction of months detected across the entire time series (2014–2019), and found that temporal occupancy at the annual and time‐series scale were strongly positively correlated (see Supporting Information for details).

### 2.4 Relating Spatial Occupancy, Temporal Occupancy, and Abundance

In order to characterize the relationship between species spatial and temporal occupancy (i.e., do species that occupy a greater fraction of sites also tend to occupy individual sites more frequently?), we conducted a linear regression for each year (2014–2019). To examine how spatial and temporal occupancy were respectively related to mean local abundance we conducted separate linear regressions where spatial occupancy was related to mean local abundance, and then where temporal occupancy was related to mean local abundance. For both spatial and temporal occupancy, we conducted regressions interspecifically and intraspecifically. Interspecific relationships explored the variation of occupancy and abundance between species (i.e., do species with greater occupancy tend to be more locally abundant?) by constructing regressions for each year where individual occupancy and abundance values represented different species. Intraspecific regressions captured the variation across time steps by using values of abundance and occupancy for a single species across multiple years (i.e., do the years in which a species is more widespread correspond to greater local abundance?). To ensure sufficient data for intraspecific analyses, only species that were present for at least 5 years were included, resulting in 53 species/subspecies across 40 genera of mammals for the latter analysis.

## 3 Results

### 3.1 The Relationship Between Spatial and Temporal Occupancy

**FIGURE 2:**
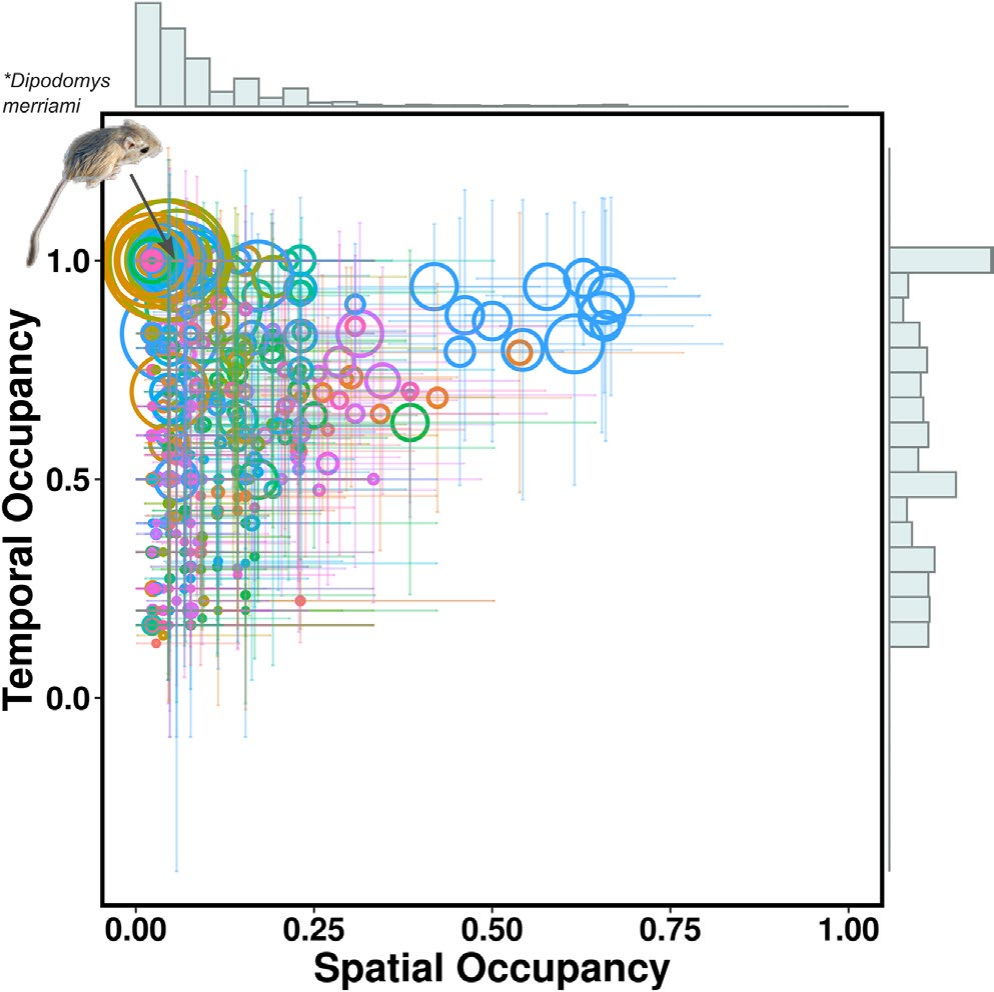
Spatial and temporal occupancy of species across years (2014−2019) are generally positively related, with some notable exceptions. Individual circles represent the spatial and temporal occupancy of each species for a given year, color represents distinct mammalian genera, and circle size the mean local abundance of that species for that year. Bars represent confidence intervals for estimates of spatial occupancy and the weighted standard deviation for estimates of temporal occupancy. Histograms represent the frequency distributions of species based on their spatial and temporal occupancy. *Dipodomys merriami* represents a persistent specialist which occupied its site(s) for 100% of the months sampled. Credit to Baiken for the photograph of *D. merriami* .

Spatial and temporal occupancy were consistently positively related across species for all years; ranging in slope between 0.39 and 0.93 with coefficient of determination (multiple *R* ^2^) values between 0.019 and 0.15 (Figure 2), and with four of the six years being statistically significant (see Supplement for yearly plots). Though it appeared that several species might be driving the observed relationships, removing *Blarina brevicauda* and members of the genus *Peromyscus*— *P. maniculatus* and *P. leucopus* , in particular—which had some of the greatest values of spatial occupancy across years (53.8%−66.7%), did not qualitatively alter the relationships, though it did reduce explanatory power and significance (see Supplement for more details).

Though spatial and temporal occupancy were positively related, they were distinctly distributed across species (Figure 2). The observed temporal occupancy values ranged from 12.5%−100% with a relatively even distribution across species (85 species occupied the landscape over 50% of the time). Spatial occupancy was more restricted and skewed towards lower values (2.27%−66.7% of sites occupied, only three species occupied more than 50% of sites). This means that multiple species had high temporal occupancy and low spatial occupancy, such as the desert rodent *Dipodomys merriami* .

### 3.2 Abundance–Occupancy Relationships Across Space and Time

**FIGURE 3:**
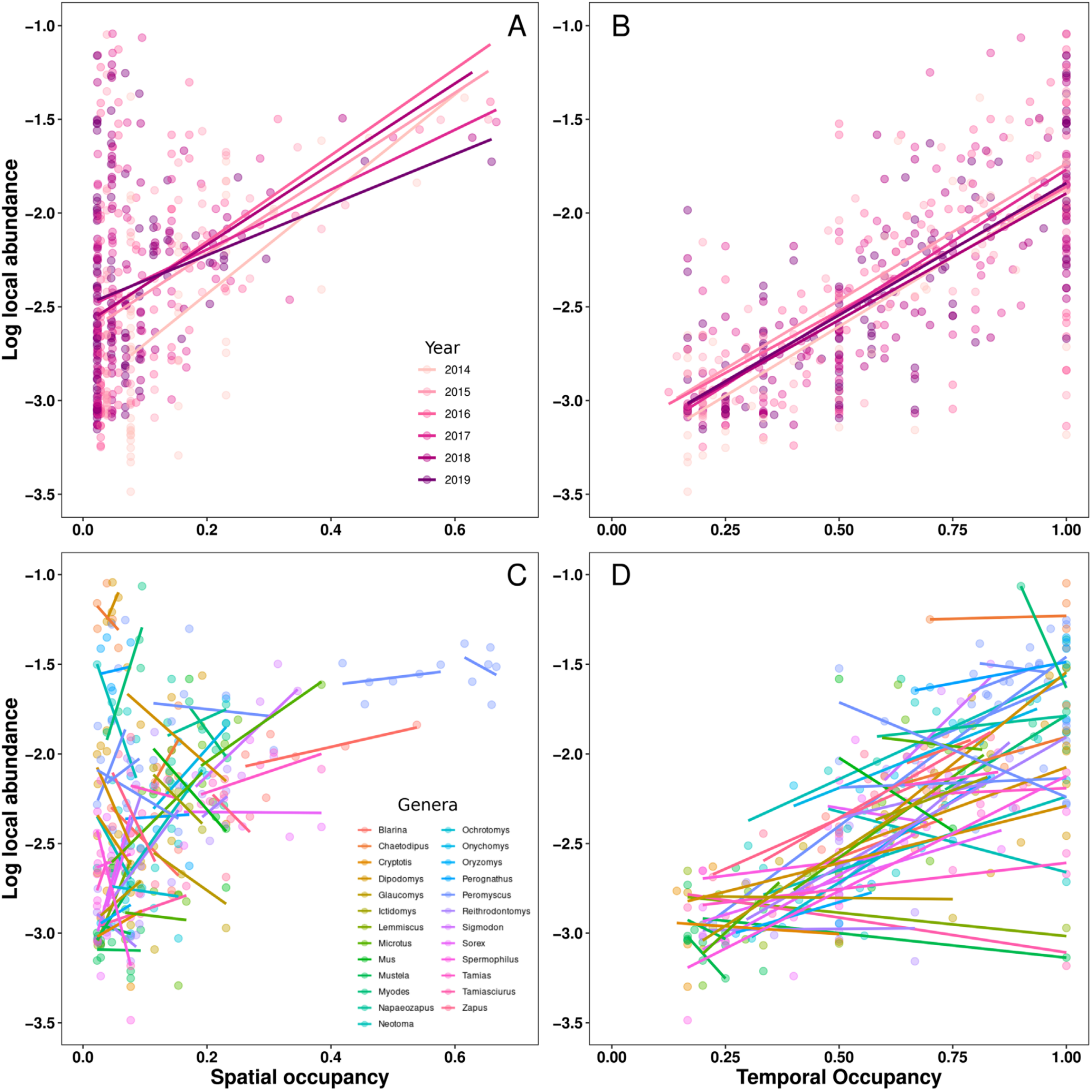
Species with greater spatial (A) or temporal occupancy (B) tended to have greater mean local abundance, and this was true across years. Within a species, however, years in which the species had high spatial (C) or temporal occupancy (D) did not necessarily correspond to greater mean local abundance. In fact, the relationship between spatial/temporal occupancy and mean local abundance tended to be nonsignificant intraspecifically. Each point represents the occupancy and abundance estimate for a species in a given year. Colors represent distinct years for interspecific relationships (A, B) and genera for intraspecific relationships (C, D).

Interspecific spatial and temporal abundance–occupancy relationships were consistently significant and positive (Figure 3). Spatial abundance–occupancy slopes ranged from 1.35 to 2.66 and resulted in multiple *R* ^2^ values between 0.06 and 0.36, depending on the year. Temporal abundance–occupancy slopes ranged from 1.32 to 1.53 and resulted in multiple *R* ^2^ values between 0.42 and 0.67. Intraspecific relationships were less consistent (Figure 3). Species spatial abundance–occupancy relationships were rather evenly divided between positive (29) and negative (24) relationships and often failed to detect an effect (49 of 53). Species temporal abundance–occupancy relationships were more positive (38 of 52) but similarly often failed to detect an effect (40 of 52). The species *Dipodomys merriami* had a positive (nonsignificant) spatial abundance–occupancy relationship but no temporal abundance–occupancy relationship as it occupied its 1−2 sites for 100% of the months sampled.

## 4 Discussion

We observed that species occupying broad spatial extents tended to occupy sites for greater portions of the year. That is, spatial and temporal occupancy were positively related. This might suggest that species with the ability to occupy a diversity of environments also have the ability to continue occupying those environments across seasons. Interestingly, few species in our dataset attained both a high spatial and temporal occupancy (i.e., widespread and persistent), rather, far more species were associated with low spatial occupancy and high temporal occupancy. These correspond to species which are nearly always present on the landscape, but are specialists to a small subset of sites studied. Understanding the tradeoffs between spatial and temporal occupancy—and the relationships of both measures of occupancy to abundance—may provide insight into habitat specialization and species persistence in competitive communities. Here, we highlight the relationships between spatial and temporal occupancy in a systematically sampled set of sites and provide a framework to help understand species occupancy in space and time.

Species geographic distributions—and resulting spatial occupancy estimates—are limited by environmental suitability, biotic interactions, and dispersal ability or geographic barriers (Pulliam 2000). Species temporal distributions are driven by environmental constraints on persistence (Tuljapurkar 2013), or, at a minimum, environmentally forced changes to species behavior (e.g., hibernation in winter). Examining the distributions of spatial and temporal occupancy estimates for species, we found that spatial occupancy estimates were right‐skewed, consistent with other systems (Heino 2008, 2015; McGeoch and Gaston 2002), with few species occupying over 50% of sampled sites. By contrast, temporal occupancy was relatively randomly distributed and departed from previous observations (unimodal (Magurran and Henderson 2003; Ulrich and Ollik 2004), right‐skewed or left skewed (Hansen and Carey 2015) and bimodal [Coyle et al. 2013]). The distinct distributions of spatial and temporal occupancy likely reflect differences in occupancy constraints, though the specific pattern observed may be sensitive to the sampling scale.

We found that spatial and temporal occupancy were positively related to local abundance across species, but less consistently related intraspecifically. This echoes previous findings that spatial abundance–occupancy relationships are positive but weak across systems (Ten Caten et al. 2022a), that intraspecific relationships tend to be weaker than interspecific relationships (Gaston et al. 1998, 1999), and that temporal occupancy is similarly related to abundance (Guo et al. 2000; Wilfahrt et al. 2021). We might expect that species with the ability to occupy diverse sites throughout the year would achieve the greatest abundances, but we observed the greatest abundances for persistent specialists (high temporal occupancy, low spatial occupancy), such as the desert rodent *Dipodomys merriami* (Reynolds 1958; Zeng and Brown 1987). If we consider temporal occupancy as a measure of habitat suitability, similar to (Coyle et al. 2013) which quantified temporal occupancy across years, then it follows that high temporal occupancy would be associated with greater local abundance, even more so than the ability to occupy a diversity of sites. In the context of abundance–occupancy relationships, this may be a potential mechanism by which outliers of the spatial abundance–occupancy relationship may be exhibiting higher than expected levels of abundance. As noted above though, these relationships are observed across a heterogeneous region, and abundance–occupancy relationships can be particularly sensitive to the spatial sampling scale (Steenweg et al. 2018) and definitions of occupancy (Ten Caten et al. 2022b). The effects of various scales on spatial and temporal occupancy are fruitful avenues for future research, but are beyond the scope of our study which aimed to understand the relationship between regional spatial/temporal occupancy and abundance.

Temporal occupancy as we define it here differs from previous explorations of species interannual persistence (Coyle et al. 2013; Hansen and Carey 2015; Snell Taylor et al. 2020; Wilfahrt et al. 2021), but see (Boss and Da Silva 2014). Investigating temporal occupancy at the intra‐annual scale captures the finer temporal dynamics of how species occupy sites. Though the qualitative results observed here were not sensitive to the annual boundaries imposed upon the time series (see Supporting information for details), the role of phenology in temporal occupancy merits further research and is known to influence support for abundance–occupancy relationships (Steenweg et al. 2018; Dallas et al. 2019). Further, quantifying temporal occupancy across a region rather than at a site captures how species tend to persist across sites, rather than capturing context‐dependent dynamics. This allows species to be considered along a spatiotemporal spectrum of occupancy.

Taken together, our results demonstrate the positive relationship between spatial and temporal occupancy, the importance of temporal occupancy in understanding patterns of abundance, and the significance of considering occupancy perspectives at the same scale, such that they can be interpreted within a unified framework. Our study builds on seminal works (Hanski 1982; Hanski and Gyllenberg 1993; Magurran and Henderson 2003) and previous efforts (Soininen and Heino 2005) in order to explore these occupancy measures in small mammals across the United States. By incorporating a spatiotemporal view of abundance–occupancy relationships, we add to an existing body of literature calling for the incorporation of temporal perspectives on ecological relationships (Wolkovich et al. 2014), including species abundance distributions Magurran (2007), ecological networks (Blonder et al. 2012), and species extinction risk (Wilfahrt et al. 2021).

## Author Contributions

**Lauren A. Holian:** conceptualization (lead), data curation (lead), formal analysis (lead), investigation (lead), methodology (lead), visualization (lead), writing – original draft (lead), writing – review and editing (lead). **Cleber Ten Caten:** conceptualization (supporting), formal analysis (supporting), investigation (supporting), methodology (supporting), writing – review and editing (supporting). **Tad A. Dallas:** conceptualization (supporting), formal analysis (supporting), funding acquisition (lead), investigation (supporting), methodology (supporting), resources (lead), supervision (lead), writing – review and editing (supporting).

## Funding

This work was supported by the National Science Foundation (NSF‐DEB‐2017826 and NSF‐DEB‐2420769).

## Conflicts of Interest

The authors declare no conflicts of interest.

## Supporting information


**Supplemental materials:** Further information on the spatial distribution of NEON sampling sites and a rigorous exploration of alternative modeling approaches.

## Data Availability

R code and data are available on figshare at https://doi.org/10.6084/m9.figshare.26060407.
