# Supplementary material for: Abundance–Occupancy Relationships are Informed by Species Temporal Occupancy
Source: Ecol Evol. 2026 Apr 6;16(4):e73214. doi: 10.1002/ece3.73214 (PMC13052264; doi:10.1002/ece3.73214)
Supplement: Supplementary file 1 — Supplemental materials: Further information on the spatial distribution of NEON sampling sites and a rigorous exploration of alternative modeling approaches. [file ECE3-16-e73214-s001.docx]

**Appendix 1 - Overview of model selection for predicting the duration of LRMs**


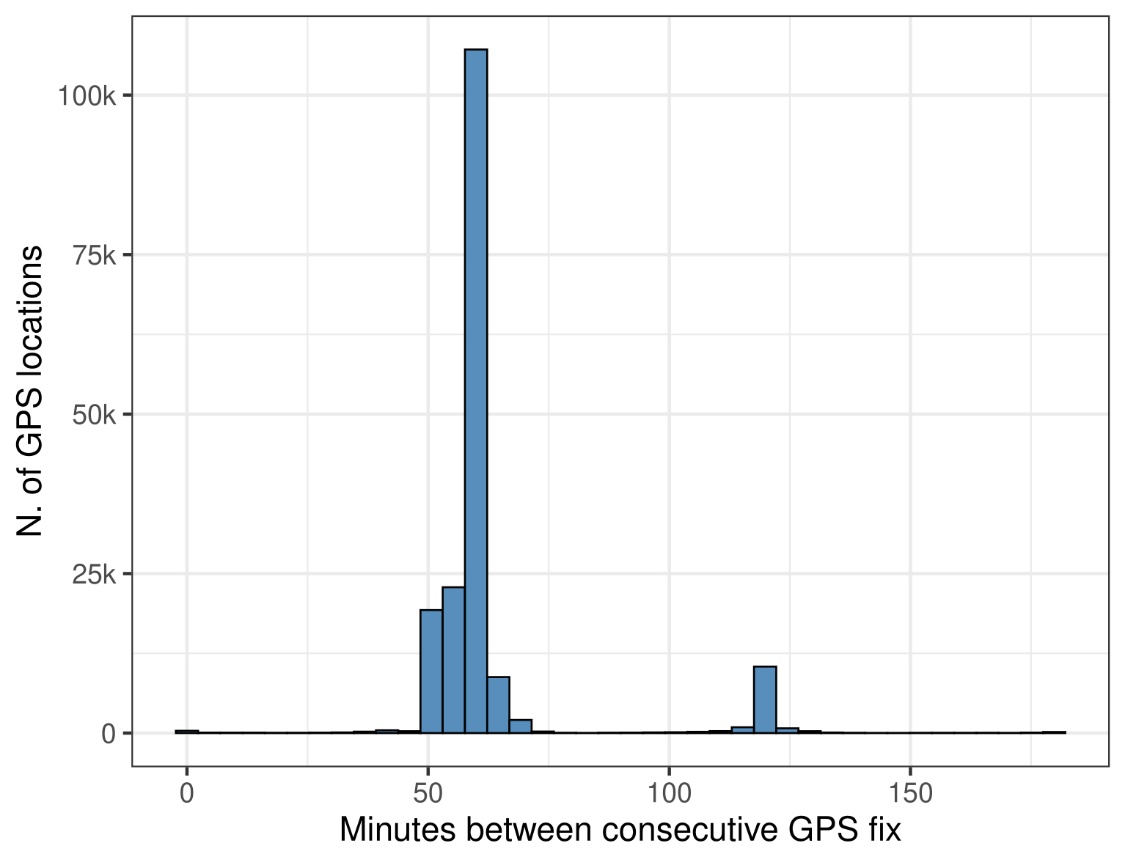


Fig. S1. Distribution of the time elapsed between consecutive GPS locations. Most GPS locations were collected every 50-70 minutes (left peak), but some every 110-125 minutes (right peak) due to irregularities connected with solar radiation and battery life.


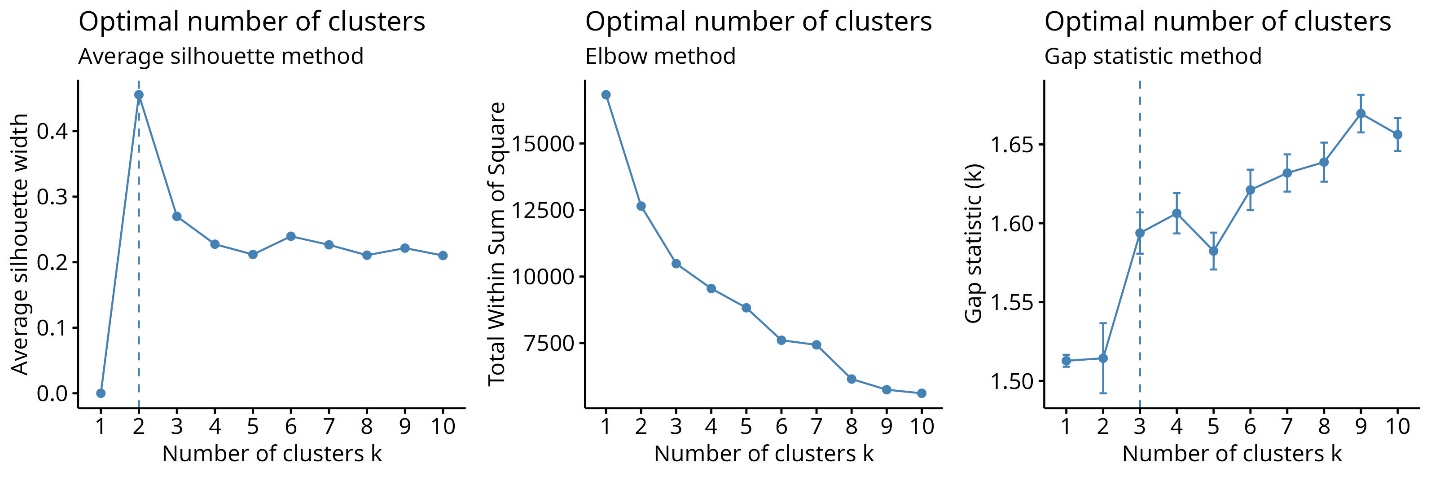


Fig. S2. Overview of the average silhouette method, the elbow method and the gap statistics method, to identify the optimal number of cluster for PAM cluster analysis. A complete description of the three methods is available in Kassambara (2017).


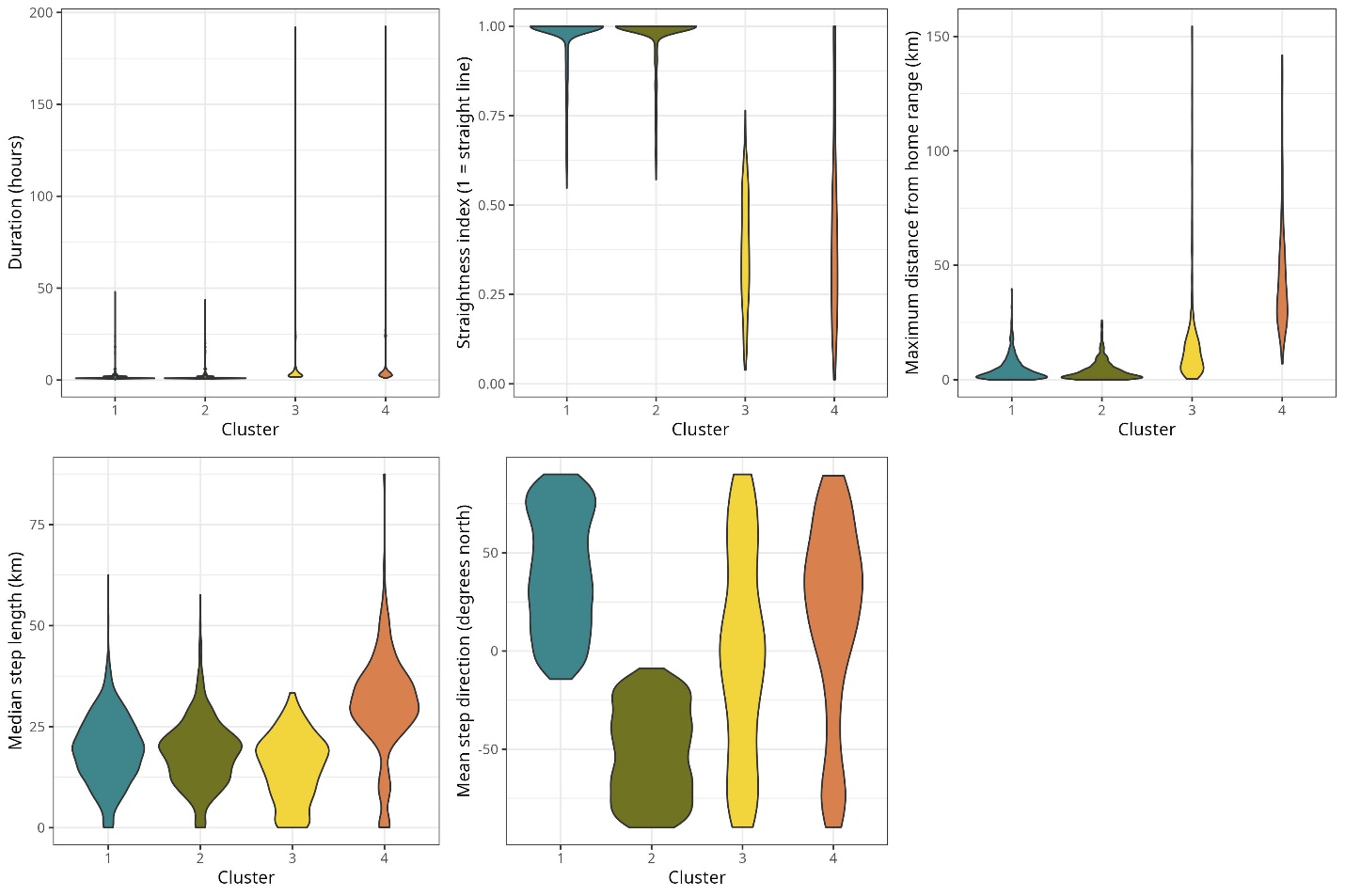


Fig. S3. Comparison between the four groups of extra-home range movement trajectories, identified by PAM cluster analysis. The four groups are compared in terms of their duration, the tortuosity, the maximum distance, the median step length and their mean step direction.


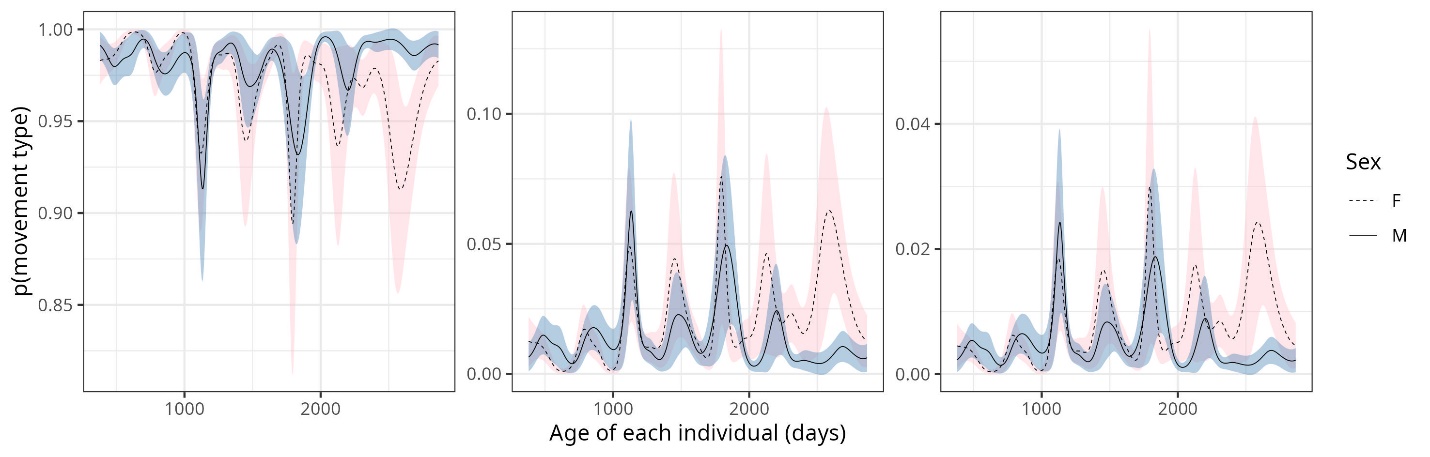


Fig. S4. Marginal effect plot, showing the effect of the age of each individual over the probability that male and female Griffon Vultures engaged in short-range (left), medium-range (middle) and long-range movements (right).


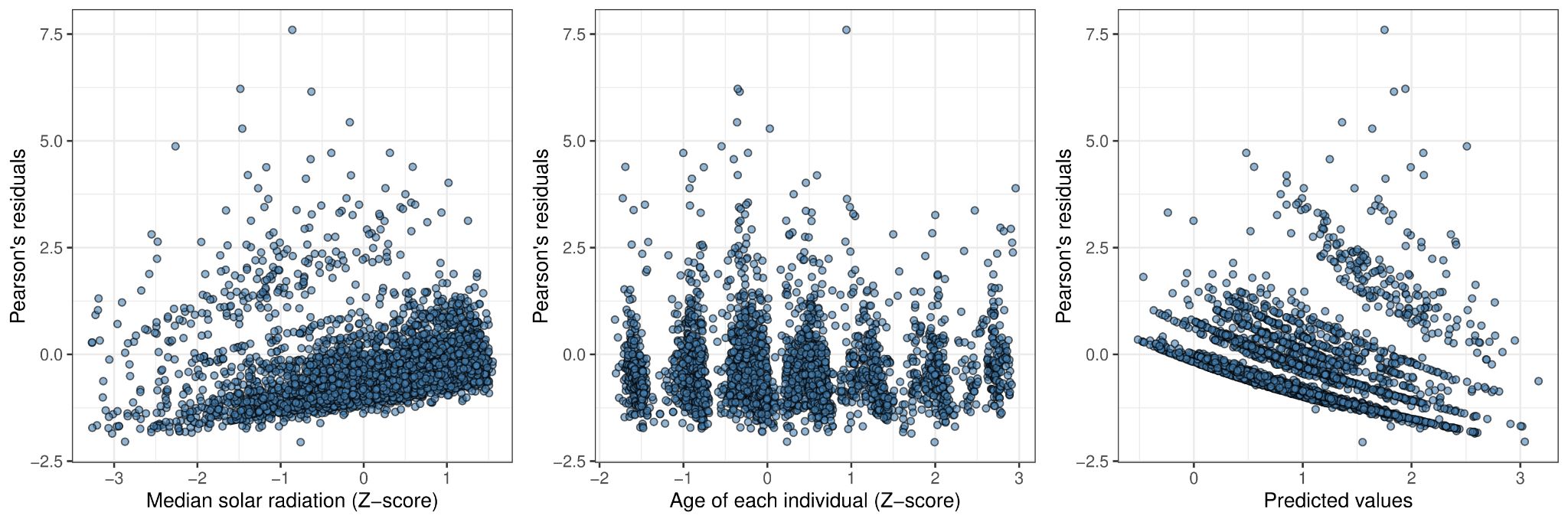


Fig. S5 Residuals of the best candidate model (mod.d) versus the median solar radiation (left), the age of each individual (center) and versus predicted values (right). The median solar radiation and the age of each individual are expressed as a Z-scores.


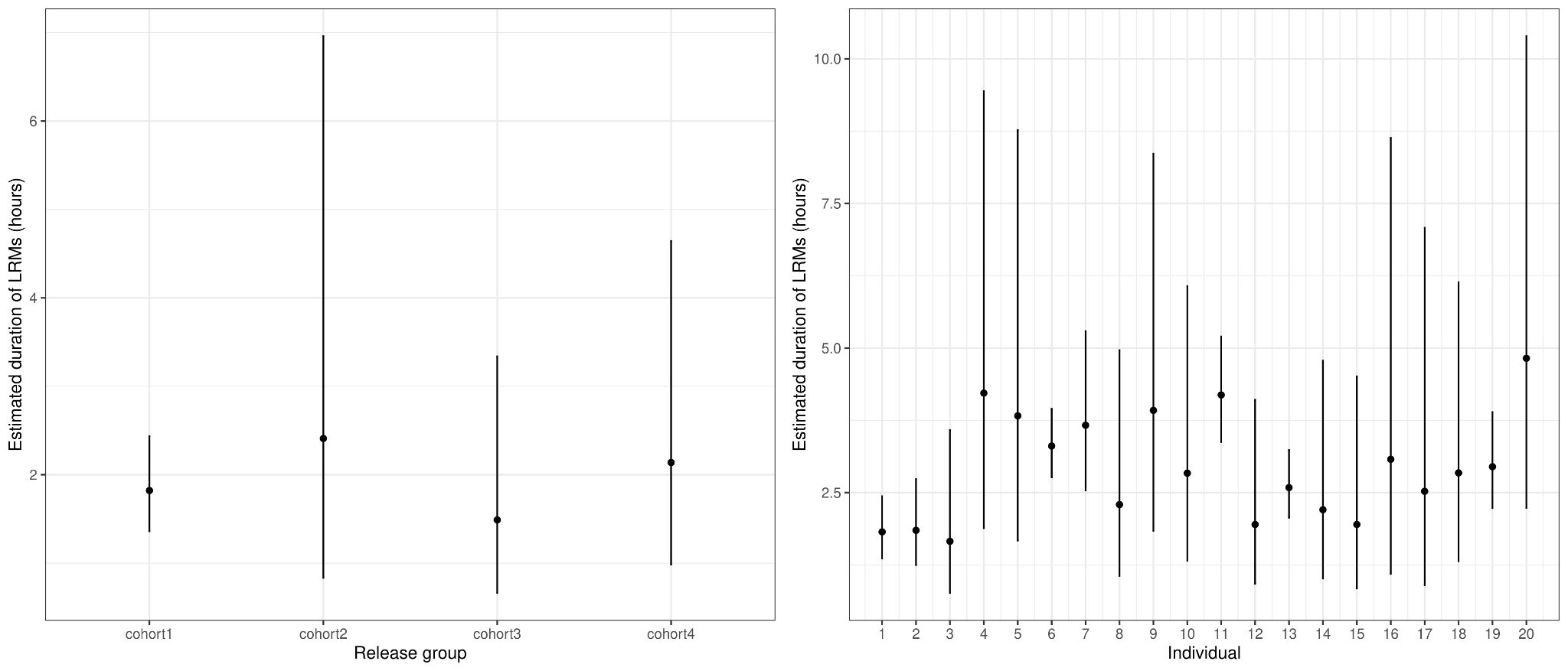


Fig. S6 Estimated duration of long-range movements (LRMs), according to the best candidate model (mod.d), between different groups of released Griffon Vultures and between different individuals


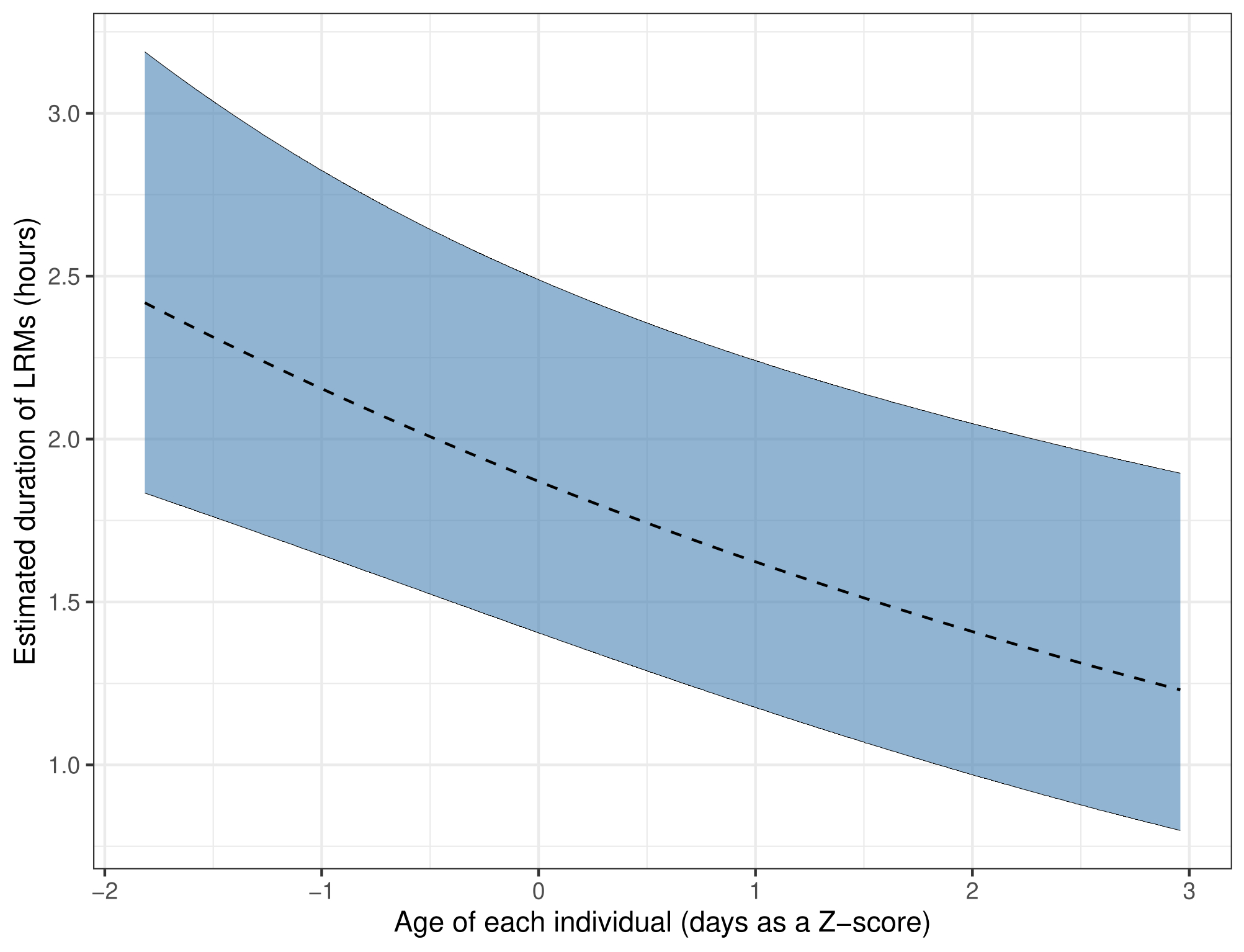


Fig. S7 Estimated duration of long-range movements (LRMs), according to the best candidate model (mod.d), based on the age of released Griffon Vultures. The age is expressed as a Z-score.

Table S1. Overview of stepwise forward model selection, representing the Akaike’s Information Criterion (AIC) and Un-biased Risk Estimator (UBRE) of each model, as well as the chi-squared and p-values of likelihood-ratio tests. Models were fitted with the “mgcv” package in R, by using a Gamma distribution of the response and Restricted Maximum Likelihood Estimation.

| **Model** | **Model structure** | **AIC** | **UBRE** | **χ^2^** | **p-value** |
| --- | --- | --- | --- | --- | --- |
| mod.null | Duration ~ 1 | 15033.0 | 1980.6 | - | - |
| mod.a | Duration ~ Cohort | 14951.2 | 1974.3 | 87.9 | p < 0.01 |
| mod.b | Duration ~ Cohort + (id, bs = “re”) | 14697.1 | 1959.5 | 283.9 | p < 0.01 |
| mod.c | Duration ~ Cohort + Age + id (bs = “re”) | 14685.3 | 1958.3 | 13.9 | p < 0.01 |
| mod.d | Duration ~ Cohort + Age + Solar radiation + id (bs = “re”) | 13629.2 | 1903.7 | 1058.3 | p < 0.01 |
| mod.e | Duration ~ Cohort + Age * Solar radiation + id (bs = “re”) | 13630.8 | 1903.2 | 0.40 | p > 0.05 |


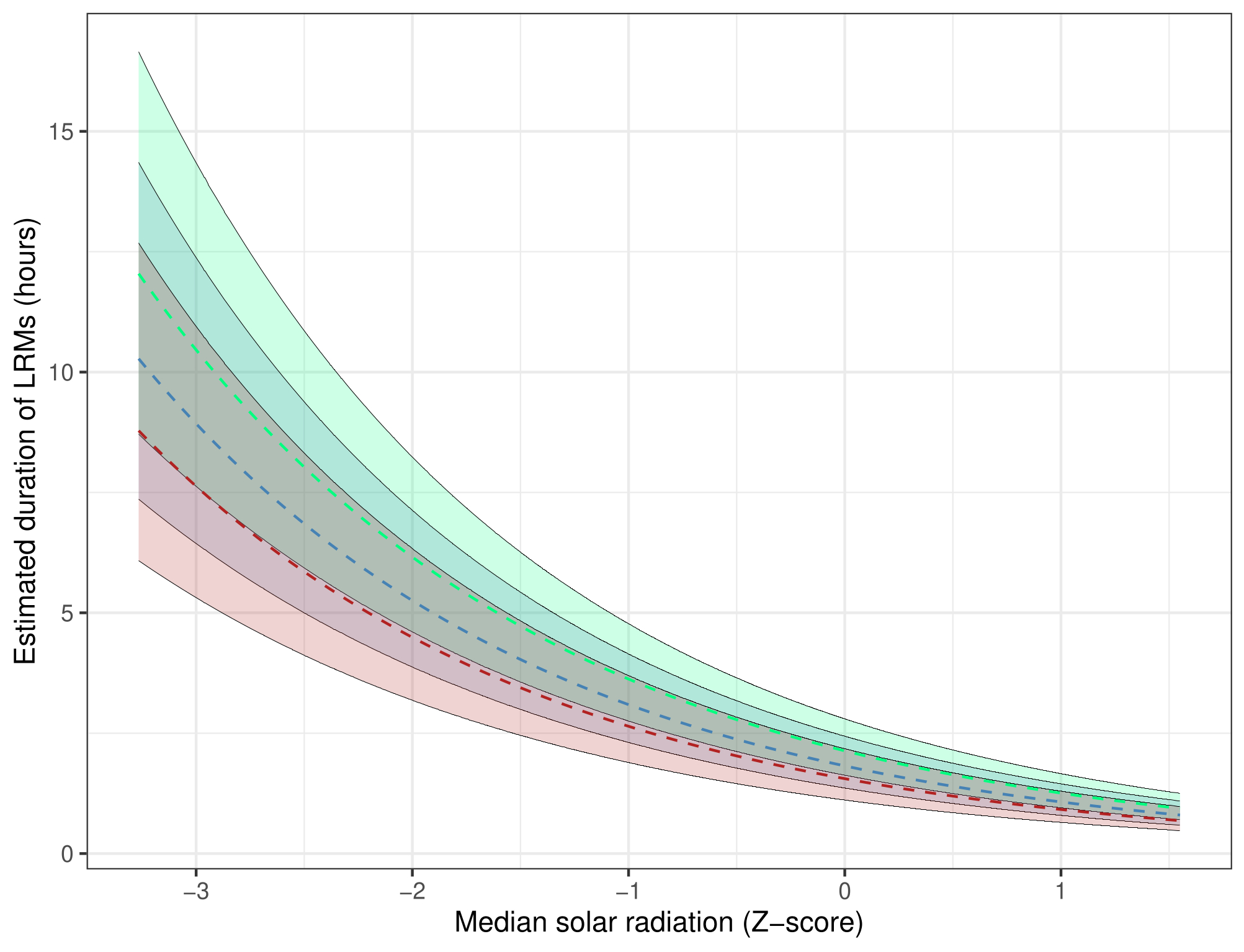


Fig. S8 Estimated duration of long-range movements (LRMs) according to the median solar radiation (as a Z-score), between Griffon Vultures of different ages. The interaction term is from “mod.e” (see Table S1) and it was not significant according to likelihood ratio test, due to its low effect size.
